# 1471. Identifying Modifiable Risk Factors for Colorectal Surgical Site Infections at a Large Safety Net Hospital: A Case-Control Study

**DOI:** 10.1093/ofid/ofad500.1307

**Published:** 2023-11-27

**Authors:** Vatsala Mundra, Bidyut Mani, Madhuri Sopirala, Roman Jandarov

**Affiliations:** University of Texas, Southwestern, Dallas, Texas; UT Southwestern Medical Center, Dallas, Texas; UT Southwestern Medical Center and Parkland Health, Dallas, Texas; University of Cincinnati College of Medicine, Cincinnati, OH

## Abstract

**Background:**

Colorectal surgical site infection (SSIs) rates have been reported to be from 10%-30% and significantly contribute to patient morbidity, mortality, and healthcare costs. High safety-net burden has been shown to be associated with increased risk of SSI, but to our knowledge, modifiable risk factors for colorectal surgery (CRS) have not been investigated in this setting. Deep and organ space infection rate at Parkland Health, a safety-net hospital in Dallas County, was 3.7% in 2021 3.6% in 2022. We aim to identify modifiable risk factors for colorectal SSI at our safety net hospital.

**Methods:**

This is a retrospective case-control study in which patients who had a deep or organ space SSI after CRS from 01/2018 to 05/2022 were randomly matched 1:3 by month. A total of 100 cases and 300 controls were studied. Data was extracted for 69 variables. All variables achieving p < 0.1 in univariate analysis were included in a multivariate logistic regression analysis.

**Table 1:**
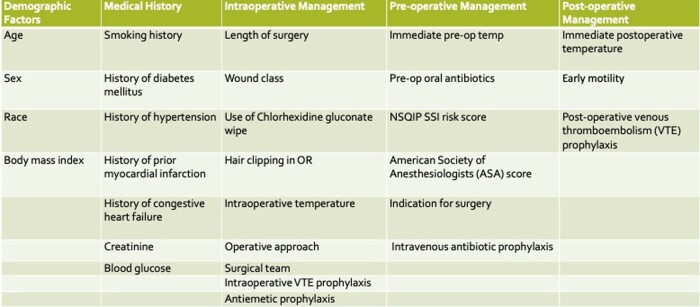
Modifiable Risk Factors. Factors regarding demographic history, medical history, intraoperative management, pre-operative management, and post-operative management were extracted and analyzed.

**Results:**

Univariate analysis was significant for: BMI18.5 - 24.9 (OR, 0.495; 95% CI, 0.274-0.895; p= 0.022) , age 40 to 49 (OR, 0.528; 95% CI, 0.287-0.970; p=0.039), early mobility (OR, 0.420; 95% CI, 0.236-0.747; p=0.003), immunosuppression (OR, 4.093; 95% CI, 1.642-10.202; p=0.003), NSQIP score of 2 (OR, 1.848; 95% CI, 1.147-2.977; p=0.016), serum glucose 200-250 (OR, 2.228; 95% CI, 1.130-4.393; p=0.032), and age 20 to 29 (OR, 2.306; 95% CI, 1.060-5.019; p=0.043). Early mobility was significant with multivariate logistic regression analysis (OR, 0.3; 95% CI, 0.14-0.66; p=0.0027).

**Figure 1.**
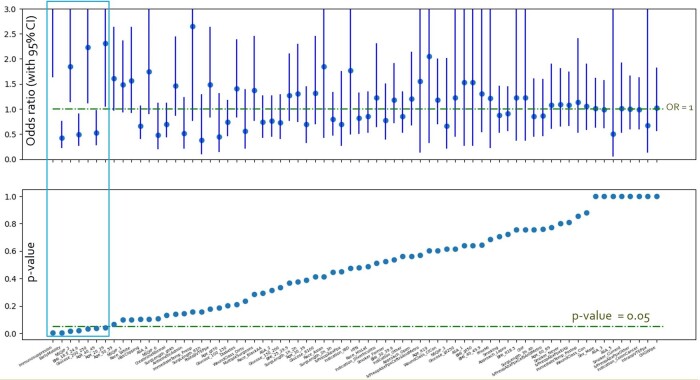
Univariate Significance Analysis of Extracted Variables

The protective factors against developing an SSI were age between 40-49, BMI between 18.5-24.5, and early mobility. The risk factors that were associated with developing an SSI were age between 20 and 29, blood glucose level between 200 mg/dL and 250 mg/dL, NSQIP score of 2, immunosuppression.

**Table 2.**
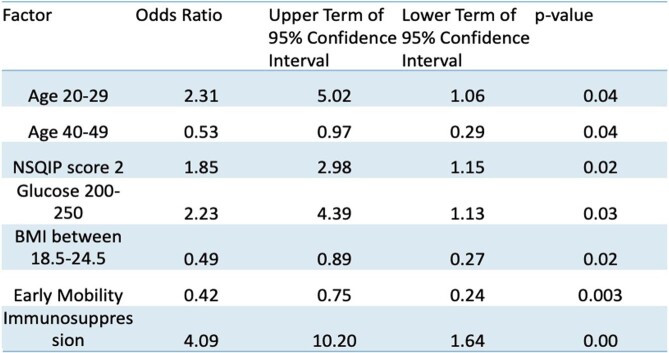
Statistical Information about Significant Variables

Seven variables were found to be significant after the completion of univariate analysis. Initial multivariate analysis indicates that early mobility may be significant with a p-value of 0.0027.

**Conclusion:**

Early mobility in the form of out of bed to chair for all meals (3 times in a day) on post-operative day one is protective against SSI occurrence. While risk factors for SSIs post-colorectal surgery have been discussed in the literature, there has not been a study conducted in a large safety net hospital that already follows the National Surgical Quality Improvement Program recommended guidelines. We were able to assess the independent importance of different variables from practice guidelines as potential risk factors in a large, diverse, safety net hospital. We engaged our surgeons and OR nursing staff in a multidisciplinary effort to optimize early mobility to reduce SSI risk.

**Disclosures:**

**All Authors**: No reported disclosures

